# Bibliometric Analysis of Dendritic Epidermal T Cell (DETC) Research From 1983 to 2019

**DOI:** 10.3389/fimmu.2020.00259

**Published:** 2020-03-12

**Authors:** Ziqin Deng, Hongwei Wang, Zhiye Chen, Ting Wang

**Affiliations:** Department of Pathogen Biology, School of Basic Medicine, Tongji Medical College, Huazhong University of Science and Technology, Wuhan, China

**Keywords:** DETC, gamma delta T cells, bibliometrics, Scopus, VOSviewer

## Abstract

Dendritic epidermal T cells (DETC) are a group of immune cells expressing canonical γδ TCR in the murine epidermis. Similar to γδ T cells in the human epidermis, DETC serve an important barrier cell in the skin and participate in skin immune surveillance, immune regulation, skin homeostasis, tissue protection, and other activities. Since its discovery in 1983, research on DETC has grown rapidly and unevenly. To evaluate DETC research trends and map the DETC knowledge structure, we have applied bibliometric methods and techniques. A total of 384 DETC-related articles obtained from the Scopus database published between 1983 and 2019 were analyzed using indicators of publication and citation metrics, country and international cooperation, author and co-authorship, and keyword co-occurrence cluster. The present research status, the emerging global trends and the future development direction are also visualized and discussed. In summary, this study provides novel and useful data for the DETC research scientific community, and will help researchers explore DETC more intuitively and effectively.

## Introduction

Dendritic epidermal T cells (DETC or DETCs), which are restricted to the murine epidermis and bear a canonical γδ TCR, are the first T cells to develop in the embryonic thymus (1). As one of the constituent cells that act as an important barrier in the skin, DETC are involved in skin homeostasis, immune surveillance, immune regulation, protection of tissues, and other physiological and pathological activities, and also play an important role in pathogen invasion resistance, wound healing, malignancy, and autoimmune diseases (2, 3).

**Figure 1:**
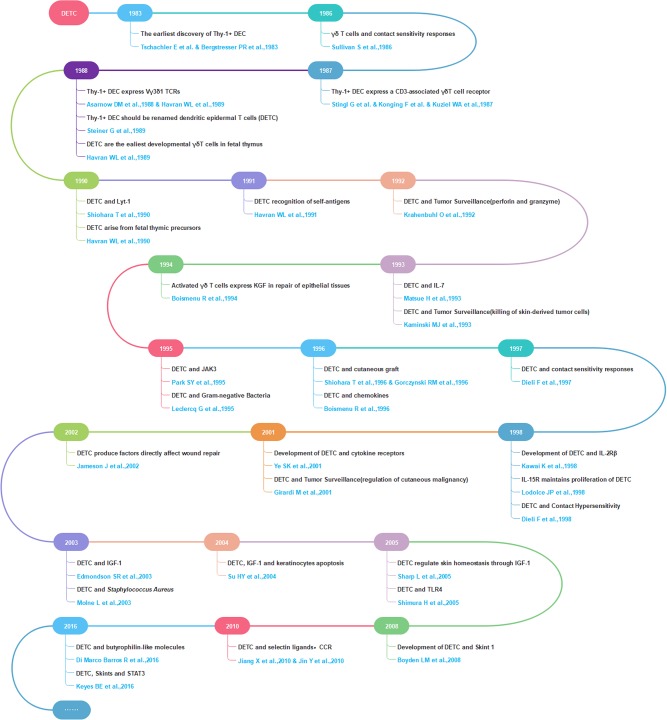
Selected milestones in DETC research.

Research on DETC has been ongoing for nearly four decades, with the important milestones for the field shown in Figure 1. Since Tschachler et al., and Bergstresser et al., simultaneously first encountered previously unrecognized Thy-1^+^ dendritic epidermal cells within murine epidermis, named Thy-1^+^ EC or Thy-1^+^ DEC (4, 5) in 1983, an increasing number of studies have been performed to better understand the characteristics and functions of Thy-1^+^ dendritic epidermal cells, which were renamed DETCs in 1988 (6). In 1987, Stingl et al., Konging et al., and Kuziel et al., found that Thy-1^+^ DEC express a CD3-associated γδ T cell receptor (7–9). Subsequently, additional research has led to a more clear understanding of DETC, including on expressing a canonical Vγ3^+^ Vδ1^+^ TCR [Garman nomenclature (10); alternative Heilig and Tonegawa nomenclature, Vγ5^+^ Vδ1^+^ (11)] (12, 13); important cell surface markers (6); DETC development, maturation, migration, and participating related receptors and ligands (1, 14–19); and the connections between DETC and epidermal homeostasis (20–24), inflammation (25–27), microbial infection (28–31), contact hypersensitivity (32–35), transplantation (36–38), tumor surveillance (39–41), and wound healing (42, 43). Hence, DETC are also considered to be a potentially ideal model for studying skin-resident γδ T cells in humans and other mammals.

To date, DETC have attracted considerable attention from various research fields, and in turn, publications on DETC have increased rapidly. However, few studies have focused on understanding DETC research in a comprehensive manner through longitudinal and global perspectives. Therefore, it is necessary and essential to apply a scientific and advanced information visualization method to reveal the progress and trends in DETC research.

Bibliometric analysis, a novel and scientific information visualization method used worldwide to evaluate the knowledge structure, development, and trends within a topic both quantitatively and qualitatively, is now widely used in a variety of fields (44, 45). The use of bibliometric analysis is still in the early stage in the immunology field and not many articles using it have been published outside of the tumor immunotherapy, microbiome, sepsis, and vaccine fields (46–49). Here, we apply a bibliometric analysis of DETC research publications (prior to September 17 2019) in terms of numbers of publications and citations, journal, country, and international cooperation, author and co-authorship and keyword co-occurrence cluster. Our aims are to help more scientists in related areas gain a better understanding of the past, present, and future of DETC, leading to more efficient and effective exploration of the frontiers, hotspots, and trends of DETC research.

## Methods

### Source Database

The publication search was performed using Scopus (https://www.scopus.com/), one of the most comprehensive databases of scientific peer-reviewed literature that includes different types of documents together with sufficient relevant information essential to our study (45).

### Search Design

We decided to search for documents related to DETC research of all types by their titles, abstracts, and keywords. In order to cover as many results that met our requirements as possible, the search query string was designed as described below.

After studying all our references, we found that DETC was first discovered as dendritic cells expressing Thy-1 antigen in murine epidermis (4, 5). After that, as further research was conducted, these cells were also commonly referred to by their cellular classification as Vγ3^+^ Vδ1^+^ T cells (Garman nomenclature) or Vγ5^+^ Vδ1^+^ T cells (Heilig and Tonegawa nomenclature) that exist in the skin of mice and rats (10, 11), or even γδ T cells located in murine epidermal in many articles.

Considering various wording and writing formats, we also studied the rules that govern the Scopus search system. All words in a term automatically include both their plurals and spelling variants (if any) at the same time and return the results altogether. Furthermore, lowercase and uppercase letters are blended when searching, and a Greek alphabet is recognized the same as its English spelling form (only when it appears alone, uncombined with other letters or words). All punctuation, hyphens, and more than one blank in a term are ignored (as one blank) in both terms and results, which means the use of alternative forms such as “Vγ3-Vδ1”, “Vγ3/Vδ1”, “Vγ3 Vδ1”, and “Vγ3^+^ Vδ1^+^” will not make a difference, but the combination caused by deletion between words like “Vγ3Vδ1” and the insertion within a word like “Vγ-3 Vδ-1” will yield different results. We found that the harmonization does not deal with a Greek letter or its English spelling form combined with other letters or words (Vγ3 and Vgamma3 will not be harmonized as the same, for example), and plurals of an abbreviation (such as DETCs) are not automatically included. Thus, we had to list all these possible situations as exhaustively as we could think of in the search formula.

According to Scopus rules, phrases enclosed in double quotes are marked as a whole (ex, “dendritic epidermal T cell”) in which words must appear together in the exact order while Boolean operators AND and OR are used to combine separate words depending on certain logical relationship. Moreover, operators (enclosed in brackets) are processed following the precedence order of OR (must contain at least one of the terms) prior to AND (both terms must be contained whenever they appear separately or together) when searching. Wildcard (^*^), like when used in ^*^epiderm^*^ for example, will return words such as epidermal, epidermis, intraepidermal, etc.

Search terms about Vγ3^+^ Vδ1^+^ T cell, Vγ5^+^ Vδ1^+^ T cell, γδ T cell, and Thy-1^+^ dendritic cell need to be confined to work in the skin or epidermis of mice or rats. To do this, qualifiers were used, with more information provided in Table 1. In addition, Skint1, a gene recently identified as essential for DETC development, was added as a search term for results related to the selection and upkeep of the cell (14, 50).

**Table 1:** Collocation of qualifiers and subjects in the search formula.

**Qualifier^a^**	**Subject^b^**
(Not applicable)	**Dendritic epidermal T cell** (“dendritic epidermal T cell” OR “dendritic epidermal T lymphocyte” OR DETC OR DETCs OR “epidermal dendritic T cell” OR “epidermal dendritic T lymphocyte”)
*epiderm* OR **skin** OR **cutaneous**	**Vγ3** OR **Vγ5** OR **Vγ3Vδ1** OR **Vγ5Vδ1** (Vγ3 OR Vgamma3 OR “V γ 3” OR “Vγ 3” OR “Vgamma 3” OR Vγ5 OR Vgamma5 OR “V γ 5” OR “Vγ 5” OR “Vgamma 5” OR Vγ3Vδ1 OR Vgamma3Vdelta1 OR “Vγ3 Vδ1” OR “Vgamma3 Vdelta1” OR “V γ 3 V δ 1” OR “Vγ 3 Vδ 1” OR “Vgamma 3 Vdelta 1” OR “V γ 3V δ 1” OR “Vγ 3Vδ 1” OR “Vgamma 3Vdelta 1” OR Vγ5Vδ1 OR Vgamma5Vdelta1 OR “Vγ5 Vδ1” OR “Vgamma5 Vdelta1” OR “V γ 5 V δ 1” OR “Vγ 5 Vδ 1” OR “Vgamma 5 Vdelta 1” OR “V γ 5V δ 1” OR “Vγ 5Vδ 1” OR “Vgamma 5Vdelta 1”)
*epiderm* OR (**skin** AND *epitheli*) OR (**cutaneous** AND *epitheli*)	**γδ** **T cell** (“γδ T cell” OR “γδ T lymphocyte” OR “gammadelta T cell” OR “gammadelta T lymphocyte” OR “γ δ T cell” OR “γ δ T lymphocyte”)
**Thy** 1 OR **Thy1**	(*epiderm* AND **dendritic**) OR (**skin** AND **dendritic**) OR (**cutaneous** AND **dendritic**) OR **DEC** OR **DECs**
(Not applicable)	**Skint1** OR **Skint 1**

a *Subjects with no qualifier to collocate with are tagged as Not applicable*.

b *For some subjects, contents enclosed in brackets are directly extracted from the search formula to list all the possible wording and writing formats used in it*.

Ultimately, the search formula used was as follows: TITLE-ABS-KEY (“dendritic epidermal T cell” OR “dendritic epidermal T lymphocyte” OR DETC OR DETCs OR “epidermal dendritic T cell” OR “epidermal dendritic T lymphocyte”) OR TITLE-ABS-KEY (^*^epiderm^*^ OR skin OR cutaneous AND Vγ3 OR Vgamma3 OR “V γ 3” OR “Vγ 3” OR “Vgamma 3” OR Vγ5 OR Vgamma5 OR “V γ 5” OR “Vγ 5” OR “Vgamma 5” OR Vγ3Vδ1 OR Vgamma3Vdelta1 OR “Vγ3 Vδ1” OR “Vgamma3 Vdelta1” OR “V γ 3 V δ 1” OR “Vγ 3 Vδ 1” OR “Vgamma 3 Vdelta 1” OR “V γ 3V δ 1” OR “Vγ 3Vδ 1” OR “Vgamma 3Vdelta 1” OR Vγ5Vδ1 OR Vgamma5Vdelta1 OR “Vγ5 Vδ1” OR “Vgamma5 Vdelta1” OR “V γ 5 V δ 1” OR “Vγ 5 Vδ 1” OR “Vgamma 5 Vdelta 1” OR “V γ 5V δ 1” OR “Vγ 5Vδ 1” OR “Vgamma 5Vdelta 1”) OR TITLE-ABS-KEY (^*^epiderm^*^ OR (skin AND ^*^epitheli^*^) OR (cutaneous AND ^*^epitheli^*^) AND “γδ T cell” OR “γδ T lymphocyte” OR “gammadelta T cell” OR “gammadelta T lymphocyte” OR “γ δ T cell” OR “γ δ T lymphocyte”) OR TITLE-ABS-KEY (Thy1 OR “Thy 1” AND (^*^epiderm^*^ OR skin OR cutaneous AND dendritic) OR DEC OR DECs) OR TITLE-ABS-KEY (“skint1” OR “skint 1”) AND (LIMIT-TO (LANGUAGE, “English”)). The limitation, (LIMIT-TO (LANGUAGE, “English”)), at the end of the formula was added in the data filtration process (explained in greater detail in section Data Filtration).

### Data Collection

All results were searched on Scopus using the formula above and exported together with as much relevant information as possible in CSV format. The search was performed all within 1 day, September 17, 2019, in case of changes brought about by update of the database.

### Data Filtration

**Figure 2:**
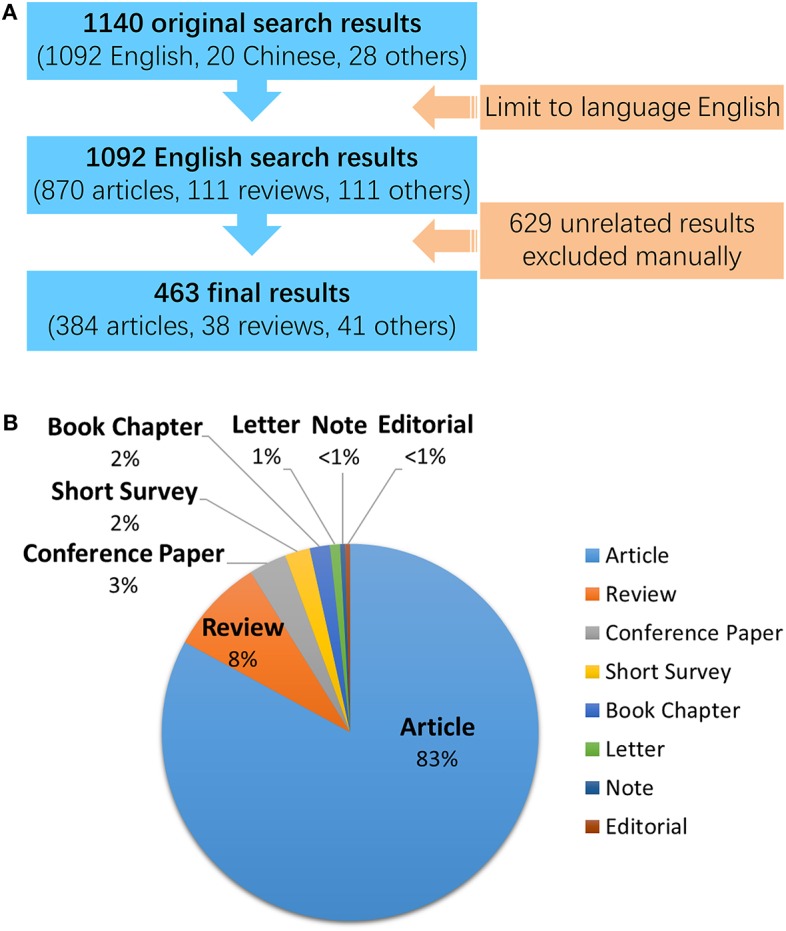
Data filtration processing and results. A total of 1,140 results of all document types and languages were found on Scopus using the original search formula given in this article. **(A)** Details of the data filtration process. A language filter was applied to the original search on Scopus so that only English results were exported regardless of their document types. Then, a manual review was performed on the exported results to filter out documents with definite relevance to DETC research. **(B)** Document types of all 463 English results identified for DETC. The pie chart illustrates the proportion of document number of each type.

Only results of documents published in English were exported. This was accomplished by directly limiting the results to English only when searching. Then, manual filtration was performed by viewing each title, abstract, keywords, and full text, to judge whether there was a clear correlation with DETC in this content. A total of 629 unrelated results were excluded among the 1,092 exported results, most of which were on γδ T cells but not in murine or rat epidermis, while others are articles that came up in the results due to multiple meanings that are sometimes ascribed to acronyms, such DETC(s), in the search terms. In total, 463 results were identified as valid after comparing and discussing both outcomes of filtration made by two authors independently (Figure 2A, Supplementary Table 1).

As shown in the pie chart in Figure 2B, most of the publications are articles, making up 83% (384) of the total; this is followed by reviews, which, at 8%, make up almost half of the remaining portion. Other types of documents, in order of quantity, were conference papers (15, 3%), short surveys (10, 2%), book chapters (8, 2%), letters (4), notes (2), and editorials (2), each of which made up only a small amount of the total, with no more than 4% individually. The following analysis was restricted to articles only (Supplementary Table 2).

### Data Analysis

The filtered database file was imported into Microsoft Excel 2016 for analysis, and included the following information: authors, title, year of publications, source journals, affiliations, citations, DOI, and keywords. GraphPad Prism 8 was also applied to create charts.

In addition to many of the self-explanatory indicators used in this paper, we employed four specialized metrics (extracted from Scopus): CiteScore, SJR, SNIP, and h-index. Each metric is explained below.

CiteScore is a new metric of journal citation impact powered by Scopus. CiteScore is the number of citations received by a journal in 1 year to documents published in the 3 previous years, divided by the number of documents indexed in Scopus published in those same 3 years (45).

Calculated by SCImago Lab and developed from Scopus data, SJR (SCImago Journal Rank) measures weighted citations received by a serial. The value of a citation is based on the subject field, quality, and reputation of the citing serial (51).

SNIP (Source Normalized Impact per Paper) was created by Professor Henk Moed at the Centre for Science and Technology Studies (CTWS), University of Leiden. It measures contextual citation impact by weighting citations based on the total number of citations in a subject field. The effect of a single citation is given a higher value for subject areas where citations are less likely and vice versa. Unlike the well-known journal impact factor, SNIP corrects for differences in citation practices between scientific fields, thereby allowing for more accurate inter-field comparisons of citation impact. CWTS Journal Indicators also provide stability intervals that indicate the reliability of the SNIP value of a journal (52).

H-index, proposed by Hirsch (53), attempts to measure both the productivity and impact of the published work of a scientist, journal, or country. The value of the index is h if a measured object has h papers that have received at least h citations each, while the rest of the papers have no more than h citations each.

### Visualization Maps

Visualization tools, like VOSviewer, Citespace, Bicomb, and BibExcel, enable researchers to create knowledge maps, evaluate the latest research progress, and identify hotspots in a research field (54). Co-authorship, co-citation, and co-occurrence analyses are the most frequently used methods. In our research, we use country co-authorship, author co-authorship, author co-citation, and author keyword co-occurrence analyses. Country co-authorship analysis provides information about collaborative relationships between authors in various countries. Cooperation preferences of authors from various countries can be used to improve cooperation with foreign authors. Author co-authorship analysis reveals collaborative relationships between authors, which can help researchers understand the relationships between researchers in a field and identify potential collaborators. For example, if some authors appear together often, they may have a closer relationship than others. Author co-citation analysis looks for a co-citation relationship between two papers, both of which are cited by a third article. Citations can provide important insight into what is already known; thus, this relationship can help identify outstanding authors. This can help new researchers to better understand the basics and progress within the field, and identify research hotspots, and other bibliometric information. Author keyword co-occurrence analysis helps researchers identify central issues and developments in a subject. Frequent co-occurrence of two keywords in an article normally suggests a closer relationship between them than other keywords, which may inspire a new research idea. In addition, it can prompt trending author keywords of each year (45, 55).

Bibliographic database files were imported into VOSviewer 1.6.12 to build network visualization maps of scientific publications, scientific journals, researchers, research organizations, countries, keywords, or terminology. Items in these networks can be connected by co-authors, co-occurrences, citations, bibliographic couplings, or co-citation links. Finally, co-authorship, co-citation, and co-occurrence analyses are presented in the form of network visualization maps.

### Research Ethics

The study was conducted as a bibliometric analysis. All data sources were available on the Internet; thus, no animal or human subject was involved, eliminating the need for permission from an ethics committee.

## Results

### Analysis of the Number of Publications and Citations

**Figure 3:**
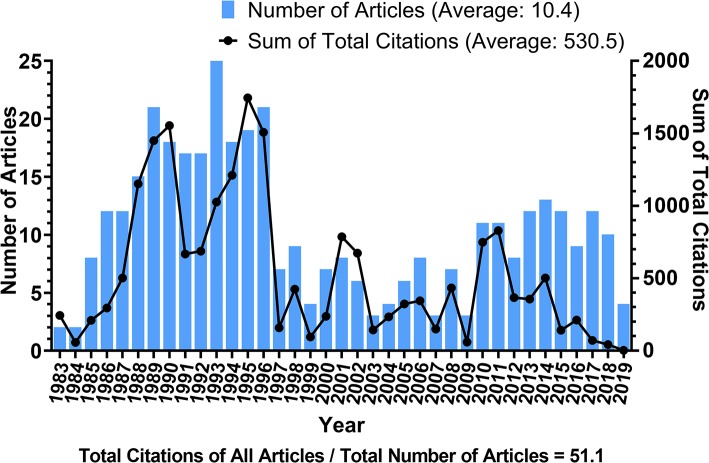
Number of articles published each year and the sum of total citations of annual articles related to DETC research. Blue bars indicate the number of articles published each year; the black line indicates the sum of total citations that annual articles have received.

The number of articles published annually and the summed total citations of annual articles related to DETC research are shown in Figure 3. Similar trends can be seen from both sets of data, although the one for annual publications is more regular and can be easily divided into three stages (Stage 1, from 1983 to 1996; Stage 2, from 1997 to 2009; Stage 3, after 2010). It is interesting to note that the line for summed citations changes approximately in synchrony with the annual publication number.

Stage 1 lasts for approximately 14 years, during which the amount of annual publications roughly increases over the years, forming a positively sloped line. Since its discovery in 1983 (4, 5), the number of articles published on DETC increased rapidly, and quickly reached 15 publications in 1988, implying that researchers were quite sensitive to the emergence of a new field. Moreover, the corresponding citation line in this stage shows two peaks, with the highest one (1,745 citations) in 1995, which was slightly delayed compared to the publication peak in 1993 (25 articles).

After the explosion of Stage 1, however, both metrics showed a sudden and significant fall in 1997. Since then, the annual publication number has remained below 10 throughout the entirety of Stage 2. During this stage, the average annual publication (5.8 articles) was less than a half of it was in Stage 1 (14.8 articles).

In Stage 3, there was a slight uptick in the number of annual publication, fluctuating right above 10 for the majority of the period. Total citation of these articles is declining, which we hypothesize to be mainly a matter of time, as these articles are newly published. It should be noted that the statistics for 2019 are incomplete, but we predict that it will still follow this pattern of Stage 3 for years to come. However, there still exists the possibility for a new phase of significant development, like what was observed in Stage 1. It is likely that, with the accumulation of steady progress and technological advances, this field is likely to take another leap forward in the near future.

**Figure 4:**
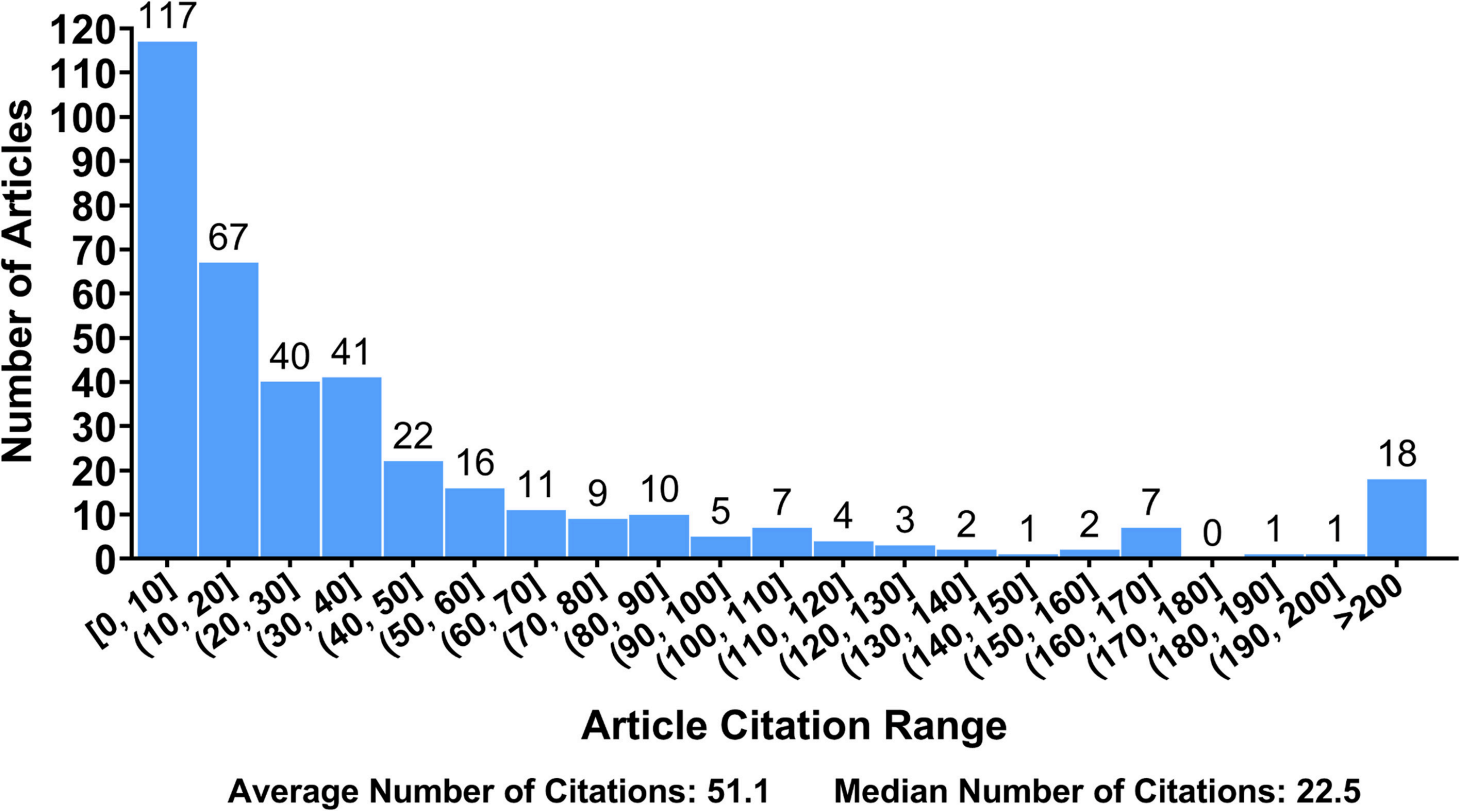
Distribution of article citation numbers. The range of citation numbers (from 0 to 759) was divided into intervals of equal width, with the exception of those articles receiving over 200 citations. The number of articles in each interval was counted.

By dividing the total citations of all articles by the total publication number, we found that each article was cited an average of 51.1 times. Figure 4 shows a histogram depicting the distribution of citations across DETC articles. The frequency declines so sharply as the number of citations increases that a severely differentiated distribution can be readily observed. One hundred seventeen articles (30.5%) are cited fewer than 10 times each. Among them, 68 articles have been cited fewer than five times each, including 12 articles that have gone uncited. Eighteen articles (4.7%) have been cited more than 200 times each, spanning a wide range of citation counts from 211 to 759. This histogram indicates that there could be many underutilized sources of information; thus, their potential value needs to be sought out.

The top 15 most highly cited articles are shown in Table 2. Twelve articles were published before 2000 (including 9 in the 1990s and 3 in the 1980s), while the remaining three were published later. Further analysis revealed that all 15 articles were published in only eight journals, all of which are in the top 20 journals for publishing the highest number of DETC articles (Table 3), and some of the authors can also be found among the list of the most productive authors (Table 5). Most of the articles in this table focused on the development, migration, regulation, or surface markers of DETC. Since these are the most frequently cited articles, we can conclude that these subjects attract a significant amount of attention within this field. Unfortunately, as we discussed above, quite a few publications have not been fully utilized, including articles on less popular subjects. We believe that this underutilization will decrease with the integration of knowledge processing, as the whole field advances in size and scope.

**Table 2:** Top 15 DETC-related articles with the most citations.

**Rank^a^**	**Title**	**Total Citations**	**Authors^b^**	**Source**	**Year**	**References**
1	Defective lymphoid development in mice lacking expression of the common cytokine receptor γ chain	759	Cao X., Shores E.W. et al.	*Immunity*. Volume 2, Issue 3, Pages 223–38.	1995	(56)
2	Regulation of cutaneous malignancy by γδ T cells	673	Girardi M., Oppenheim D.E. et al.	*Science*. Volume 294, Issue 5542, Pages 605–9.	2001	(39)
3	Migration and maturation of langerhans cells in skin transplants and explants	541	Larsen C.P., Steinman R.M. et al.	*Journal of Experimental Medicine*. Volume 172, Issue 5, Pages 1483–93.	1990	(57)
4	Modulation of epithelial cell growth by intraepithelial γδT cells	530	Boismenu R., Havran W.L.	*Science*. Volume 266, Issue 5188, Pages 1253–5.	1994	(42)
5	Junctional sequences of T cell receptor γδ genes: Implications for γδ T cell lineages and for a novel intermediate of V-(D)-J joining	452	Lafaille J.J., DeCloux A. et al.	*Cell*. Volume 59, Issue 5, Pages 859–70.	1989	(58)
6	A role for skin γδ T cells in wound repair	405	Jameson J., Ugarte K. et al.	*Science*. Volume 296, Issue 5568, Pages 747–9.	2002	(43)
7	Homing of a γδ thymocyte subset with homogeneous T-cell receptors to mucosal epithelia	405	Itohara S., Farr A.G. et al.	*Nature*. Volume 343, Issue 6260, Pages 754–7.	1990	(59)
8	Developmental defects of lymphoid cells in Jak3 kinase-deficient mice	404	Park S.Y., Saijo K. et al.	*Immunity*. Volume 3, Issue 6, Pages 771–82.	1995	(27)
9	Limited diversity of γδ antigen receptor genes of thy-1^+^ dendritic epidermal cells	400	Asarnow D.M., Kuziel W.A. et al.	*Cell*. Volume 55, Issue 5, Pages 837–47.	1988	(12)
10	A role for endogenous transforming growth factor β1 in Langerhans cell biology: The skin of transforming growth factor β1 null mice is devoid of epidermal Langerhans cells	397	Borkowski T.A., Letterio J.J. et al.	*Journal of Experimental Medicine*. Volume 184, Issue 6, Pages 2417–22.	1996	(60)
11	Developmentally ordered appearance of thymocytes expressing different T-cell antigen receptors	392	Havran W.L., Allison J.P.	*Nature*. Volume 335, Issue 6189, Pages 443–5.	1988	(1)
12	IL-17 is essential for host defense against cutaneous *Staphylococcus aureus* infection in mice	343	Cho J.S., Pietras E.M. et al.	*Journal of Clinical Investigation*. Volume 120, Issue 5, Pages 1762–73.	2010	(28)
13	Recognition of self-antigens by skin-derived T cells with invariant γδ antigen receptors	302	Havran W.L., Chien Y.-H., Allison J.P.	*Science*. Volume 252, Issue 5011, Pages 1430–2.	1991	(21)
14	Identification and induction of keratinocyte-derived IL-10	287	Enk A.H., Katz S.I.	*Journal of Immunology*. Volume 149, Issue 1, Pages 92–95.	1992	(61)
15	Interleukin 7 receptor-deficient mice lack γδ T cells	258	Maki K., Sunaga S. et al.	*Proceedings of the National Academy of Sciences of the United States of America*. Volume 93, Issue 14, Pages 7172–7.	1996	(62)

a *Ranked by total citations*.

b *Names of the first two authors (on Scopus) were provided*.

**Table 3:** Top 20 journals with the most DETC-related articles published.

**Rank^a^**	**Journal**	**Articles**	**Total citations^b^**	**Citations per article**	**CiteScore 2018^c^**	**SJR 2018^c^**	**SNIP 2018^c^**	**Publisher**
1	*Journal of Investigative Dermatology*	78	2,406	30.8	3.47	1.893	1.287	Elsevier
2	*Journal of Immunology*	63	3,010	47.8	4.41	2.521	1.040	American Association of Immunologists
3	*European Journal of Immunology*	22	641	29.1	3.83	2.046	0.992	Wiley-Blackwell
4	*Journal of Experimental Medicine*	15	1,846	123.1	9.83	7.941	2.143	Rockefeller University Press
5	*Proceedings of the National Academy of Sciences of the United States of America*	15	1,205	80.3	8.58	5.601	2.539	National Academy of Sciences
6	*International Immunology*	10	179	17.9	4.25	2.082	1.191	Oxford University Press
8	*Immunity*	8	1,834	229.3	14.69	11.299	3.851	Elsevier
7	*Nature*	8	1,662	207.8	15.21	16.345	9.199	Springer Nature
9	*Immunology*	8	277	34.6	3.99	1.607	0.986	Wiley-Blackwell
10	*Science*	7	2,280	325.7	15.21	13.251	7.311	American Association for the Advancement of Science
11	*PLoS ONE*	7	148	21.1	3.02	1.100	1.123	Public Library of Science
12	*Journal of Dermatological Science*	7	61	8.7	2.63	1.235	1.178	Elsevier
13	*Frontiers in Immunology*	7	57	8.1	4.71	2.021	1.092	Frontiers Media S.A.
14	*British Journal of Dermatology*	6	129	21.5	2.45	1.984	1.714	Wiley-Blackwell
15	*Journal of Leukocyte Biology*	6	73	12.2	3.69	1.929	1.003	Wiley-Blackwell
16	*Cell*	5	1,004	200.8	24.38	25.976	6.570	Elsevier
17	*Nature Immunology*	5	624	124.8	14.71	13.300	4.302	Springer Nature
18	*Carcinogenesis*	5	77	15.4	4.33	1.820	1.064	Oxford University Press
19	*Cellular Immunology*	5	75	15.0	3.13	1.275	0.808	Elsevier
20	*Journal of Clinical Investigation*	4	451	112.8	10.49	7.001	2.462	American Society for Clinical Investigation

a *Ranked by article number. Journals with the same number of articles were then ranked by total citations*.

b *Total citations refer to the sum of citations that articles related to DETC research published in each journal have totally received*.

c *CiteScore, SJR (SCImago Journal Rank), AND SNIP (Source Normalized Impact per Paper) are metrics extracted from Scopus, introduced in detail in the Data Analysis section above*.

Again, it is important to be aware that citation does not perfectly reflect the quality of an article (particularly for new publications or publications in areas that are less popular during particular time period), nor is it the only measure. Regardless, we suggest an increased amount of multidisciplinary work and the discovery of new applications for DETC will allow each article to be fully recognized for its achievements.

### Analysis of Journal

The top 20 journals, as ranked by number of articles related to DETC research, are listed in Table 3. We used this metric instead of using the total citations of these articles for the reasons mentioned above, though this information is shown in Table 3, along with the CiteScore and SJR from Scopus as an indicator of impact. For authors, articles related to DETC research are more likely to be accepted by these journals, as they have previously shown significant interest in publishing DETC-related work. Some of the listed journals, like *Frontiers in Immunology, Nature Immunology*, and *PLoS ONE*, are newly founded, and have shown passionate interest in DETC since their establishment.

Although not shown in the table, we found that *Journal of Investigative Dermatology*, which ranks at the top of the list, is almost the only journal that published DETC-related articles prior to 1986 and published the first articles declaring the discovery of DETC (4, 5). While the 20 journals listed cover 76% of DETC articles overall, this journal alone contributes 20% of the publications.

### Analysis of Country and International Cooperation

Articles analyzed were produced by 25 countries from around the world (articles co-authored by more than one country are counted repeatedly by VOSviewer). However, most are concentrated in the United States, which contributed 205 articles, exceeding the average of all 25 countries (19.3) by a significant amount. Unlike the journal analysis (Table 3) discussed above, citations per article did not vary much between countries, with an average of 49.6 for all 25 countries, although gaps in publications and total citations are still significant between some countries (Table 4). This may have something to do with the scientific and economic environment in each country, as well as the access to international resources.

**Table 4:** Top 20 countries with the most DETC-related articles.

**Rank^a^**	**Country**	**Articles**	**Total citations^b^**	**Citations per article**	**Rank^a^**	**Country**	**Articles**	**Total citations^b^**	**Citations per article**
1	United States	205	13,156	64.2	11	France	7	306	43.7
2	Japan	75	2,469	32.9	12	Netherlands	7	265	37.9
3	United Kingdom	37	3,087	83.4	14	Sweden	5	143	28.6
4	Germany	27	755	28.0	13	Singapore	4	178	44.5
5	Austria	22	878	39.9	15	Poland	4	22	5.5
6	Switzerland	18	983	54.6	16	Israel	3	112	37.3
7	Australia	18	651	36.2	17	Denmark	3	50	16.7
8	Canada	13	313	24.1	18	Finland	2	62	31.0
9	China	12	131	10.9	19	Italy	2	42	21.0
10	Belgium	10	225	22.5	20	India	2	24	12.0

a Ranked by article number. Countries with the same number of articles were then ranked by total citations.

b *Total citations refer to the sum of citations that the articles have totally received*.

We cannot ignore the fact that the ranking has been performed using articles only published in English, which means there are quite a few articles in other languages that have not been counted (shown in Figure 2A). If added, they might make a significant difference in the ranking; as China (20 search results in Chinese) and Russia (14 search results in Russian) in particular could have the potential to rank higher. Therefore, it seems that more work should be done on document translation to promote the sharing of resources and information, which will provide many benefits to researchers.

**Figure 5:**
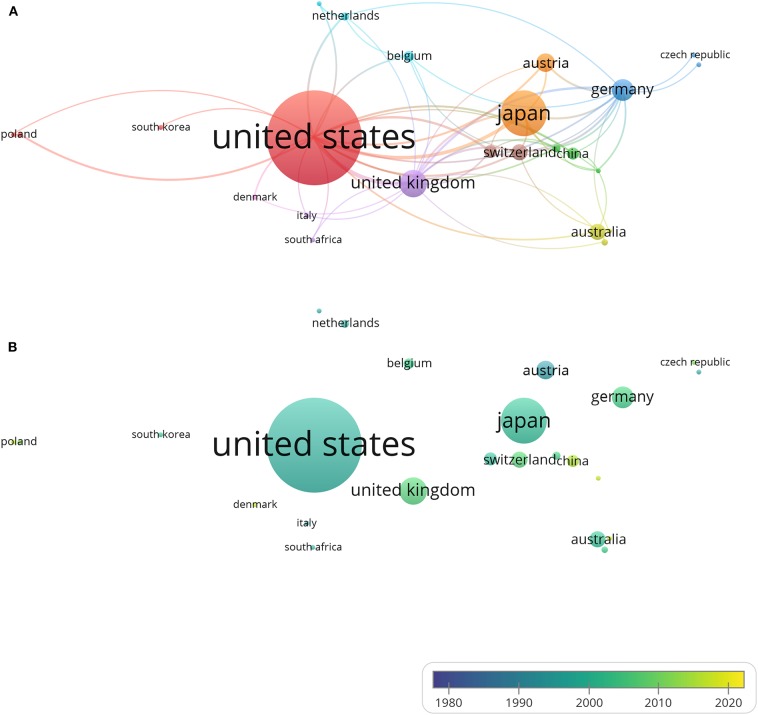
Country co-authorship analysis. The size of each circle indicates the number of articles produced by that country. The distance between any two circles indicates the relatedness of their co-authorship link, and the thickness of the connecting line indicates the strength of the link. **(A)** Country co-authorship network visualization map. The color of each circle indicates the cluster it was grouped into by VOSviewer. **(B)** Country co-authorship overlay visualization map. The color of each circle indicates the average publication year for the country, according to the color gradient shown in the lower right corner.

We used VOSviewer to analyze the co-authorship between different countries and produced two international co-authorship (international cooperation) visualization maps of publications for countries that have published more than one document. Country co-authorship maps can help researchers to understand existing partnerships and identify potential collaborators. The largest set of connected countries consists of 25 countries in nine clusters. Figure 5A illustrates the country co-authorship network. Clusters are grouped by the frequency of shared co-occurrence terms that represent each country. Closely related terms are grouped into the same cluster with the same color. The more publications a country has produced, the larger the size of its circle will be; the larger the scale of the cooperation is, the thicker the connecting line will be. For example, the link strength (a measure of collaboration) between United States and United Kingdom is 13, and they are connected by a thick line. The United States has the highest degree of cooperation with other countries, with a total link strength of 58. Researchers from the United Kingdom and Japan collaborate the most with American researchers. Figure 5B depicts the country co-authorship overlay. The color of a country is based on its average publication year. The United States, for example, with an average publication year of 1997.8 for its 205 articles, is shown in cyan. This does not mean that the United States issued the most documents in 1997 though; in fact, the United States published documents every year for the period from 1983 to 2019, making its average publication year 1997.8. As can be seen from the figure, Austria is blue (with an average publication year of 1992.8), indicating that Austrian researchers were more active at the start of DETC research; China, Singapore, and Denmark are yellow (with average publication years of 2013.7, 2015.3, and 2015.7, respectively), indicating that they are newly active in this field. The differences in the average publication years for DETC between countries and regions reflect their uneven scientific levels. National development strategies and initiatives in these countries in science and technology are enabling a strengthening of their scientific practices.

### Analysis of Author and Co-authorship

A total of 1,394 authors are involved in the articles analyzed here. The 21 most productive authors are listed in Table 5 (with two authors tied for 20th). Articles drafted by multiple authors are counted multiple times by VOSviewer. The author affiliations listed in this table are the latest one shown on Scopus and are only used to distinguish different people.

**Table 5:** Top 20 authors with the most DETC-related articles.

**Rank^a^**	**Author**	**Articles**	**Total citations^b^**	**Citations per article**	**Affiliation^c^**	**H-index^d^**
1	Tigelaar Robert E.	29	2,735	94.3	Yale Skin Diseases Research Core Center, London, United Kingdom	47
2	Bergstresser Paul R.	27	1,148	42.5	UT Southwestern Medical School, Dallas, United States	55
3	Havran Wendy L.	26	3,095	119.0	Scripps Research Institute, San Diego, United States	41
4	Takashima Akira	23	1,020	44.3	University of Toledo College of Medicine, Toledo, United States	49
5	Stingl Georg	20	860	43.0	Medizinische Universitat Wien, Vienna, Austria	79
6	Hayday Adrian C.	18	1,986	110.3	The Francis Crick Institute, London, United Kingdom	73
7	Elbe Adelheid	17	494	29.1	Medizinische Universitat Wien, Vienna, Austria	17
8	Kripke Margaret L.	14	381	27.2	University of Texas MD Anderson Cancer Center, Houston, United States	61
9	Allison James P.	11	1,721	156.5	University of Texas MD Anderson Cancer Center, Houston, United States	114
10	Halliday Gary Mark	11	257	23.4	Royal Prince Alfred Hospital, Sydney, Australia	50
11	Lewis Julia M.	10	1,424	142.4	Yale University School of Medicine, New Haven, United States	22
12	Tschachler Erwin	10	595	59.5	Medizinische Universitat Wien, Vienna, Austria	60
13	Shevach Ethan M.	10	455	45.5	National Institute of Allergy and Infectious Diseases, Bethesda, United States	97
14	Kawai Kazuhiro	10	218	21.8	Kido Hospital, Niigata, Japan	24
15	Yokoyama Wayne	9	527	58.6	Washington University School of Medicine in St. Louis, St Louis, United States	79
16	Witherden Deborah A.	9	474	52.7	University of California, San Diego, San Diego, United States	21
17	Girardi Michael	8	1,359	169.9	Yale University School of Medicine, New Haven, United States	35
18	Jameson Julie Marie	8	484	60.5	California State University San Marcos, San Marcos, United States	22
19	Coligan John E.	8	445	55.6	National Institute of Allergy and Infectious Diseases, Bethesda, United States	68
20	Plum Jean R.	8	211	26.4	Universiteit Gent, Ghent, Belgium	40
21^e^	Leclercq Georges	8	211	26.4	Universiteit Gent, Ghent, Belgium	33

a *Ranked by article number. Authors with the same number of articles were then ranked by total citations and h-index*.

b *Total citations refer to the sum of citations that each author's articles related to DETC research have totally received*.

c *Affiliation for each author is the latest one shown on Scopus, in order to distinguish different people only*.

d *H-Index, is extracted from Scopus, introduced in detail in the Data Analysis section*.

e *Two authors tied for the 20th with the same number of articles and citations; thus, the actual number of authors in the list is 21*.

Notably, the author with the highest h-index was awarded the Nobel Prize in Physiology or Medicine along with Tasuku Honjo in 2018, for their discovery of cancer therapy via the inhibition of negative immune regulation. Because of the hard work of the researchers in this field, and the fascinating discoveries they made, DETC has certainly become a more attractive field that many talented researchers are eager to join.

**Figure 6:**
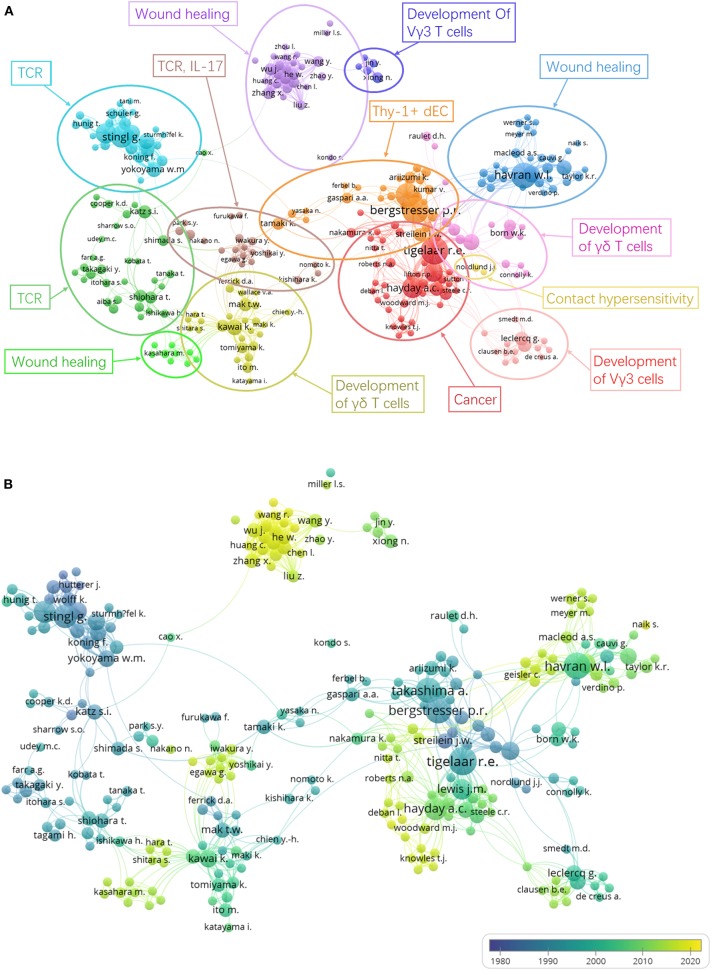
Author co-authorship analysis. Last names of authors are listed. The size of each circle indicates the number of articles produced by the author. The distance between any two circles indicates the relatedness of their co-authorship link, and the thickness of the connecting line indicates the strength of the link. **(A)** Author co-authorship network visualization map. The color of each circle indicates the cluster it was grouped into by VOSviewer. Authors are grouped manually according to the similarity of their research areas. **(B)** Author co-authorship overlay visualization map. The color of each circle indicates the average publication year for the author, according to the color gradient in the lower right corner.

For co-authorship analysis (Figure 6A), authors in a cluster represent a closely connected group and are labeled in the same color. The size of a circle represents the number of publications of an author, and the thickness of a line represents the scale of collaboration between authors. After analyzing the articles from each cluster, we manually and subjectively defined the main research area of each cluster. There still exist several independent clusters in the same area, however. As can be seen from Figure 6A, these clusters are related to “TCR” (9), “Wound healing” (43), “Cancer” (39), “Development” (63), etc. These publications are listed in our references and they have made a significant contribution to each of these research areas. Figure 6B depicts the co-authorship overlay, and can provide valuable information. Authors' colors are based on their average publication year. Havran and Hayday are shown in aquamarine on the map, indicating that they have been working on DETC since the initiation of this field. In many countries, as they become more developed economically, they also began to dedicate more attention and funding to scientific research to stay competitive or even lead the frontiers nowadays. This has led to the active participation of more scientists from a wider range of countries in recent years, as can be seen in the DETC field. These newer researchers are shown in yellow. He, the pioneer of DETC research in mainland China who has made many important contributions, is among them. It can be concluded from the figure that Chinese researchers, shown in yellow to indicate their newness, are enthusiastic and strongly connected with each other, but they are lacking the connections to researchers from other countries. We believe that the trend of increasing globalization will benefit researchers significantly and multinational cooperation will bring about unexpected changes to DETC research.

**Figure 7:**
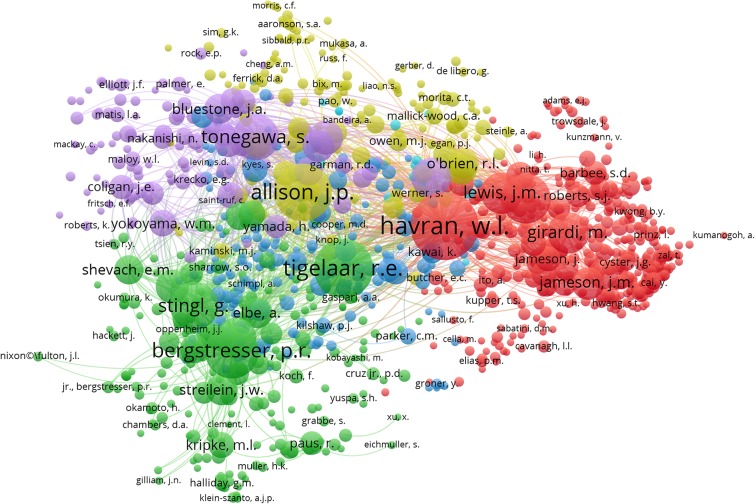
Author co-citation network visualization map. Last names of authors are listed. The size of each circle indicates the total number of citations of the author's DETC-related articles and the color indicates the cluster it was grouped into by VOSviewer. The distance between any two circles indicates the relatedness of their co-authorship link, and the thickness of the connecting line indicates the strength of the link.

In the author co-citation analysis (Figure 7), the relevance of authors is determined by the number of times that their articles are referenced by the same article. Each node represents an author with at least seven citations. The analysis includes 17,260 authors, all of whom appear in the references lists of the 384 articles that were analyzed for this paper, and 1,106 of them have published articles no fewer than seven times. The largest set of authors with the highest total link strength consisted of 1,106 people in six clusters. In this case, the same color represents authors that are within the same topic of interest, while authors in the same cluster represent a closely connected group based on co-citation relationships. Authors who are closely related often work in the same field and have contributed to the establishment and development of the field. In addition, the size of each circle represents the number of citations of the author, with more citations generating a larger circle. This is different from the perspective provided in Table 5, as this figure provides a very intuitive picture of the academic status and influence of scientists in the DETC field. Because the number of citations is related to the length of the research years and the study of certain research hotspots, young scientists who are currently in a small circle may become big players in this field in the future.

### Analysis of Keyword Co-occurrence Cluster

**Figure 8:**
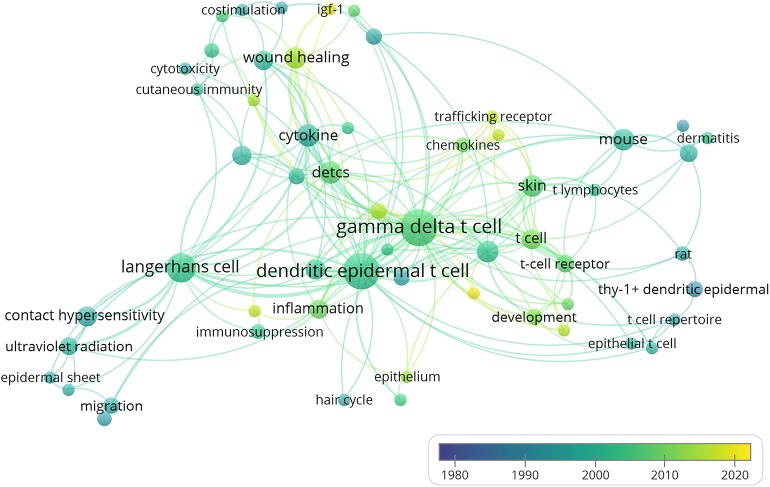
Author keyword co-occurrence analysis. Author keywords are labeled. The color of each circle indicates the average year when the keyword appeared in articles, according to the color gradient color in the lower right corner. The size of each circle indicates the frequency of occurrence of the author keyword. The distance between any two circles is indicative of their co-occurrence link, and the thickness of the connecting line indicates the strength of the link.

Keyword co-occurrence analysis details the topics covered in a DETC study with author keywords assigned to each article. Keywords are standardized texts or terms selected from the title and the text to express the subject matter of a paper, so the information can be more easily archived. Keywords provide a reasonable description of research hotspots and are very effective in bibliometric analysis when studying knowledge structures in scientific field. In the VOSviewer keyword co-occurrence visualization map, author keywords are marked in different colors according to their average publication years. For example, “Migration” (1995) and “Keratinocytes” (1995) were mainly found in the early years, in comparison with keywords such as “IL-17” (2014) and “Wound healing” (2013), which show up in more recent years (Figure 8). Keywords such as “Development,” “TCR,” “Trafficking receptor,” and “Wound healing” are yellow-green, indicating that these fields have become popular in recent years and may become hot topics in the future. In our analysis, we found that Nielsen et al. mentioned that the ligands recognized by the DETC-associated TCR are still unknown, with information such as how the DETC-Skint interaction induces and regulates the activation of DETC and how it affects wound healing still unclear. Issues like these may become popular topics for future research (64). In the DETC field, some problems remain unsettled or have only recently been undertaken, including understanding the specific molecular mechanisms of wound healing, skin immunology, transplant rejection, and microbiological interactions. However, progress is being made in these areas. High-quality articles, like the one published in 2016 by the Fuchs lab entitled “Impaired Epidermal to Dendritic T Cell Signaling Slows Wound Repair in Aged Skin,” come out every year (65). We expect there to be more breakthroughs in wound healing, skin immunology, and cell receptor function. As shown in Figure 8, research has yet to elucidate the connection between DETC and many other immune cells (NK cell, NKT cell, Th cell, CTL, ILC, B cell, myeloid cells, etc.), and therefore, we can expect to see future work dissecting these relationships, leading to many more important discoveries.

## Discussions

During this present information explosion, scientific researchers are often concerned with how to manage their knowledge. Our paper seeks to provide an innovative way to manage knowledge and visualize knowledge structures. Our study provides the most up-to-date analysis of global scientific publications related to DETC research from 1983 to 2019. This is the first report to use bibliometric indicators, visualization information tools, and techniques to reveal the knowledge map of the development, evolution, and major trends of DETC research in an intuitive manner.

The number of articles in a research area can reflect the topic's productivity and development over the years (66). The development of DETC research over the past 37 years was an uneven process, as determined in our study. The number of annual publications and citations indicate that, from 1983 to 1996, there was an upsurge period. However, later in 1997, the number of publications suddenly dropped and related progress appeared to slow. This was likely because much of the interest in immunology shifted to T cells and antiviral immunity. Despite this shift, DETC was back in the spotlight in 2010 when more in-depth studies on the pathological role that DETC plays in wound healing, cancer, infection, and inflammation were carried out. Since then, annual publication has remained at a substantial level (Figure 3). We believe the changes driven by the interplay between immunology and newer disciplines such as epigenetics and proteomics in 2010, as well as the functional characteristics of T cell subsets and hot topics like microRNA, have helped to move forward theoretical and practical research on DETC.

The number of times an article is cited reflects the extent of its dissemination and influence, and thus may partly reflect its quality as well (67, 68). According to the citation analysis (Figure 4), 30.5% of the DETC articles we analyzed have been cited no more than 10 times, indicating that there are plenty of information sources that may not be utilized to the fullest extent. On one hand, the rareness of high-impact articles may be due to the limitation of work, funds, and time for a given author; on the other hand, the lack of citations may also result from a lack of attention to some subjects within the DETC field. Regardless, such underutilization is not expected; thus, we suggest more work be done on the integration of knowledge, particularly as the DETC field continues to grow. The most cited article has acquired 759 citations thus far, and the top 9 most cited articles (cited no fewer than 400 times) were published in *Immunity* (27, 56), *Science* (39, 42, 43), *Journal of Experimental Medicine* (57), *Cell* (12, 58), and *Nature* (59), respectively (Table 2).

Popular journals and other trends in a research area during a certain period can be easily identified and can provide a reliable reference for researchers (69). Furthermore, core journals provide a significant amount of information, which is helpful, especially when searching for documents or submitting research achievements (70, 71). Among the journals in which DETC-related articles were published, the *Journal of Investigative Dermatology* has published the most articles, while articles in the *Journal of Immunology* have obtained the most total citations, and *Science* ranks first by citations per article (Table 3). Newly founded journals listed in the table, such as *Frontiers in Immunology, Nature Immunology*, and *PLoS ONE*, also deserve the attention of researchers. Their active performance indicates their potential to improve their rank with the publishing of more high-quality DETC-related articles in the future.

A number of national studies on research productivity have been conducted in recent years, primarily in order to help judge the science policy of a country and thereby adjust their science funding (72). Visualization tools like VOSviewer, Citespace, Bicomb, and BibExcel enable researchers to create knowledge maps (54). With the help of the co-authorship visualization map made by VOSviewer, a co-authorship analysis of countries was performed (45, 55, 73, 74). The map showed that the US, Japan, and the UK are the most involved in DETC research, with the US far ahead of the others, as it has been the most productive DETC research contributor since 1983. Still, with the improvement of academic standards and research funding in recent years, DETC-related studies are launching in countries including China, Singapore, and Denmark. There has been widespread international cooperation among DETC researchers, mainly led by countries like the US, Japan, and the UK (Figure 5). These developed countries have invested significant money, manpower, and material resources in scientific research. Thus, not surprisingly, they have emerged as worldwide leaders in DETC research. Meanwhile, in developing countries, especially some Asian countries like China, policies and circumstances have shifted, leading to increased support for scientific research, suggesting that these countries will become more significant players in the DETC field in the future.

In order to determine the most productive authors, we ranked authors based on their total number of DETC-related articles and performed the analysis together with other indicators to provide a more comprehensive view (75, 76). Based on the data extracted from Scopus, we found that, of the top 20 most productive authors, 12 are from the US, 3 are from Austria, 2 are from the UK, 2 are from Belgium, and the other 2 authors come from Japan and Australia (Table 5). In addition, the author co-authorship network visualization map produced by VOSviewer can also be applied to the analysis of author productivity and active period (77) (Figure 6). Notably, researchers Wendy L. Havran (3rd in Table 5) and Adrian C. Hayday (6th in Table 5) have been committed to this field from its initiation. Meanwhile, the Nobel Prize winner James P. Allison has published just 11 DETC-related articles so far, though his citation rate per article has already reached 156.5, indicating his strong influence in this area. Regardless of which aspect is evaluated (the academic level, contribution, or influence of researchers in the DETC field), the information shown in Figures 6, 7 and Table 5 all overlap to some extent, without being completely identical. This reflects dynamic changes related to the time period and hotspots in DETC research. Because of the undeniable contributions of the academic pioneers and emerging young researchers highlighted in these figures, the DETC field has been able to attract a broad group of talented researchers to join in.

In recent studies, some researchers have tried using keyword co-occurrence networks for knowledge mapping (54, 78–83). According to the VOSviewer keyword co-occurrence analysis, the early stage of DETC research was focused on the development, migration, and surface marker of these cells; then, it turned to topics such as contact hypersensitivity, ultraviolet radiation, skin cancer, immunosuppression, inflammation, cytokines, and chemokines. It appears that topics such as wound healing, skin immunology, and cell receptor function are likely to attract more attention in the future (Figure 8). Some areas of DETC biology remain unsolved or have made minimal progress, including elucidation of the specific molecular mechanisms underlying wound healing, skin immunology, transplant rejection, and microbiological interactions, as well as the interactions between DETC and other immune cells. However, our analysis reveals that scientists are still actively exploring the uncharted territory of DETC. High-quality articles published by the Hayday (84) and Fuchs labs (65) have gained significant attention and recognition in recent years. We expect to see more breakthroughs in skin immunology, immune metabolism, wound healing, and cell receptor function in the future.

Currently, there are fewer researchers involved in DETC studies than in other immune cell studies worldwide, and there still exist plenty of unresolved questions regarding DETC. We hope this bibliometric analysis provides a beneficial reference on the main points, as well as the future trends, of DETC research, not only for researchers who have already been working in this field, but also for new researchers preparing to become active members of this field.

## Data Availability Statement

Datasets generated for this study will be made available by the authors, to any qualified researcher on request.

## Author Contributions

TW designed the study. TW, ZD, and HW performed the search and wrote the paper. TW, ZD, HW, and ZC analyzed the data.

### Conflict of Interest

The authors declare that the research was conducted in the absence of any commercial or financial relationships that could be construed as a potential conflict of interest.
